# NOX2-Dependent Reactive Oxygen Species Regulate Formyl-Peptide Receptor 1-Mediated TrkA Transactivation in SH-SY5Y Cells

**DOI:** 10.1155/2019/2051235

**Published:** 2019-12-02

**Authors:** Martina Castaldo, Cristiana Zollo, Gabriella Esposito, Rosario Ammendola, Fabio Cattaneo

**Affiliations:** Department of Molecular Medicine and Medical Biotechnology, School of Medicine, University of Naples Federico II, Naples, Italy

## Abstract

Several enzymes are capable of producing reactive oxygen species (ROS), but only NADPH oxidases (NOX) generate ROS as their primary and sole function. In the central nervous system, NOX2 is the major source of ROS, which play important roles in signalling and functions. NOX2 activation requires p47^phox^ phosphorylation and membrane translocation of cytosolic subunits. We demonstrate that SH-SY5Y cells express p47^phox^ and that the stimulation of Formyl-Peptide Receptor 1 (FPR1) by N-fMLP induces p47^phox^ phosphorylation and NOX-dependent superoxide generation. FPR1 is a member of the G protein-coupled receptor (GPCR) family and is able to transphosphorylate several tyrosine kinase receptors (RTKs). This mechanism requires ROS as signalling intermediates and is necessary to share information within the cell. We show that N-fMLP stimulation induces the phosphorylation of cytosolic Y490, Y751, and Y785 residues of the neurotrophin receptor TrkA. These phosphotyrosines provide docking sites for signalling molecules which, in turn, activate Ras/MAPK, PI3K/Akt, and PLC-*γ*1/PKC intracellular cascades. N-fMLP-induced ROS generation plays a critical role in FPR1-mediated TrkA transactivation. In fact, the blockade of NOX2 functions prevents Y490, Y751, and Y785 phosphorylation, as well as the triggering of downstream signalling cascades. Moreover, we observed that FPR1 stimulation by N-fMLP also improves proliferation, cellular migration, and neurite outgrowth of SH-SY5Y cells.

## 1. Introduction

Neurotrophic factors, such as neurotrophin-nerve growth factor (NGF), brain-derived neurotrophic factor (BDNF), ciliary neurotrophic factor (CNTF), glial-derived neurotrophic factor (GDNF), and neurotrophin-3 and 4 (NT-3 and NT-4), represent a family of biomolecules necessary for neuronal survival and plasticity. They support growth and differentiation of both developing and mature neurons by binding transmembrane receptors and, in turn, stimulating protein tyrosine phosphorylations in downstream signalling cascades. Neurotrophic factors also play a key role in neurodegenerative diseases and neuropsychiatric disorders such as Bipolar Disorders (BD), depression, and eating disorders. Neurotrophins bind the tyrosine kinase receptors (RTKs) Trk; CNTF binds the CNTF receptor complex and GDNF family members signal through the tyrosine kinase receptor c-Ret [1]. NGF, BDNF, NT-3, and NT-4 also bind the p75 neurotrophin receptor (p75^NTR^), a member of the TNF receptor superfamily [2], which is a potential cell death receptor and whose activity is nullified by Trk tyrosine kinase signalling [3, 4]. Trk receptors belong to a family of three RTKs and each neurotrophin binds to a specific member of Trk family with NGF binding to TrkA, BDNF and NT-4 binding to TrkB and NT-3 binding to TrkC [5, 6].

TrkA receptor activation results in phosphorylation of Y670, Y674, and Y675 localized in the activation loop of the kinase cytoplasmic domain, which enhances tyrosine kinase activity of the receptor [5]. Y490, Y751, and Y785 of TrkA are the main phosphorylated tyrosine residues in the juxtamembrane region, in the tyrosine kinase domain, and in the intracellular C-terminal tail, respectively [5, 7]. These phosphorylated tyrosines create docking sites for the recruitment of proteins containing PTB or SH2 domains. Phosphotyrosine 490 interacts with Shc, Frs2, and other adapter molecules, which trigger the activation of Ras/MAPK and PI3K/Akt pathways. Y751 phosphorylation is essential for PI3K docking and activation, whereas phosphorylated Y785 recruits PLC*γ*1 and activates PKC [5, 7].

Formyl-peptide receptors (FPRs) belong to the G protein-coupled receptor (GPCR) family and are associated with pertussis toxin- (PTX-) sensitive Gi proteins [8]. Their main function is to detect the presence of noxious molecules and to drive cells till the site of release of harmful molecules. The sensing function of FPRs includes the detection of pathogens and of formylated peptides derived from mitochondrial peptides. Other endogenous ligands include the antimicrobial peptide LL-37, the anti-inflammatory lipid lipoxin A4, the proinflammatory molecule SAA, urokinase and its receptor, resolvins, beta amyloid (A*β*)_1-42_ peptide, prion protein (Prp)_106–126_, humanin, neuropeptides, the dual pro- and anti-inflammatory protein annexin 1, chemokine variants, vasoactive intestinal peptide, and pituitary adenylate cyclase-activating polypeptide (PACAP) [9–16].

Human FPR1, FPR2, and FPR3 are functional members of the FPR family. FPR1 binds efficiently to the formylated peptide N-formyl-methionyl-leucyl-phenylalanine (N-fMLP), whereas FPR2 is activated by WKYMVm peptide [17]. FPR3 does not bind to N-fMLP, and shares some nonformylated peptide ligands with FPR2 [18]. However, a set of formylated peptides have been identified as natural agonists for FPR3 [19]. FPR1 and FPR2 are expressed in several cell types [20, 21], whereas FPR3, is expressed in monocytes, dendritic cells [8, 16], and HUVEC cells [22]. FPR2 is also expressed on nuclear membranes of cancer cells [23], whereas FPR3 is localized within the cytoplasm [24]. Overall, binding of specific agonists to FPR1 is associated with proinflammatory responses, while FPR2 is an unusual receptor because it can convey opposite signals, depending on the ligands and on different receptor domains used by distinct agonists [25, 26]. Endocrine cells and neurons in the central nervous system (CNS) also express FPR1 [8, 27], whereas neurons of the vomeronasal organ express the other members of the FPR family [28, 29]. FPRs signalling exerts a modulatory effect on anxiety-like behaviours. In fact, *Fpr1^−/−^* mice exhibit normal spatial memory and learning capacity, but reduced anxiety-like behavior, increased exploratory activity, and impaired fear memory [30]. *Fpr2/3*-deficient mice show increased explorative behaviour and reduced fear, as well as a distinct profile of behaviour characterized by reduced anxiety [31].

RTK transactivation mediated by GPCR agonists represents a mechanism to increase the information exchange across the entire cell and to coordinate the multitude of physiological or pathological stimuli to which a cell is exposed. Several molecular mechanisms are responsible for RTK transactivation by GPCRs. They include the activation of metalloproteases, the activation of cytosolic membrane-associated tyrosine kinases, and NADPH oxidase-dependent reactive oxygen species (ROS) generation [32]. Over the last years, six homologs of the NADPH oxidase have been identified: NOX1, NOX2, NOX3, NOX4, NOX5, DUOX1, and DUOX2 and are referred as the NOX family of NADPH oxidases [33] which produces ROS as their primary and sole function. NOX2 consists of the cytosolic proteins p47^phox^, p67^phox^, p40^phox^, Rac1, and of membrane-bound subunits p22^phox^ and gp91^phox^. In the nervous system, NOX2 is the most important source of ROS and its activation requires phosphorylation and membrane translocation of the p47^phox^ subunit. In phagocytic and nonphagocytic cells, FPR stimulation by cognate agonists results in MEK- and PKC-dependent p47^phox^ phosphorylation and, in turn, in ROS generation [9, 20, 34, 35], which act as intracellular second messengers by activating several redox signalling cascades.

In different cell types, FPR stimulation induces phosphorylation of Y951, Y996, and Y1175 residues of VEGFR2 [36] and of Y1313, Y1349, and Y1356 residues of the HGF receptor [35], as well as EGFR transactivation [37], suggesting that FPRs are able to integrate GPCR signalling with tyrosine kinase activity of RTKs even in the absence of direct stimulation by growth factors [32]. ROS play a crucial role in these cross-talk mechanisms. In fact, blockade of NADPH oxidase functions by siRNA or by specific inhibitors prevents EGFR, VEGFR2, and c-Met transphosphorylation [35–37].

Trk receptors can also be activated via GPCR stimulation without the involvement of neurotrophins [38]. In human monocytes, cross talks between FPR1, EGFR, and TrkA are essential for receptor-mediated activation and to modulate proinflammatory mediators [39]. Adenosine can activate TrkA phosphorylation through an adenosine 2A receptor, a member of the GPCR family [40] and the neuropeptide PACAP, which acts through a GPCR, transactivates TrkA [41]. Furthermore, the activation of angiotensin receptor type-2 transactivates TrkB [42].

Herein, we show that the SH-SY5Y neuroblastoma cell line expresses NOX2 components, FPR1, and TrkA, and that FPR1 stimulation by formylated peptides induces NADPH-dependent ROS generation, as well as the phosphorylation of Y490, Y751, and Y785 residues of TrkA. Cytosolic phosphotyrosines of TrkA act as docking sites for signalling proteins that, in turn, activate Ras/MAPK, PLC-*γ*1/PKC, and PI3K/Akt intracellular cascades. Furthermore, FPR1-mediated TrkA transactivation promotes cell proliferation, wound healing, and neurite outgrowth of SH-SY5Y cells.

## 2. Materials and Methods

### 2.1. Reagents

SH-SY5Y cell line (ATCC, Manassas, VA, USA) was cultured in Dulbecco's modified Eagle's medium (DMEM) (Thermo Fisher Scientific, Monza, Italy) containing 15% foetal bovine serum (FBS) (Invitrogen). After reaching 80% confluence, cells were serum-starved for 24 hours and stimulated with N-fMLP (Sigma) at the final concentration of 0.1 *μ*M for 2, 5, or 10 minutes. In other experiments, serum-deprived cells were preincubated for 16 hours with pertussis toxin (PTX) (Sigma) at a final concentration of 100 ng/mL, or with 5 *μ*M cyclosporin H (CSH) (Sigma) for 30 minutes, or with 5 *μ*M rottlerin (Sigma) for 1 hour, or with 100 *μ*M apocynin (Sigma) for 2 hours, or with 10 *μ*M GW441756 (Sigma) for 1 hour, before the stimulation with N-fMLP for 5 minutes. SDS-PAGE reagents were obtained from Bio-Rad (Hercules, CA, USA). Anti-phosphoAkt(S473) (cat. no. 4060), anti-phosphoP38MAPK(T180, Y182) (cat. no. 4511), anti-CD133 (cat. no. 5860), anti-phosphoTrkA(Y490) (cat. no. 9141), and anti-phosphoTrkA(Y785) (cat. no. 4168) were from Cell Signalling Technology (Danvers, MA, USA). Anti-phosphoTrkA(Y751) (cat. no. 44-1342G) was from Life Technologies. Anti-phosphop47^phox^(S359) (cat. no. GTX55429) was from GeneTex (Irvine, CA, USA). Anti-phosphoERK 1/2 (cat. no. SC-81492), anti-PKC*α* (cat. no. SC-8393), anti-PKC*δ* (cat. no. SC-937), anti-phosphoPKC*δ* (T507) (cat. no. SC-11770), anti-ERK 1/2 (cat. no. SC-514302), ant-Akt (cat. no. SC-8312), anti-TrkA (cat. no. SC-398728), anti-tubulin (cat. no. SC-8035), anti-rabbit (cat. no. SC-2357), and anti-mouse (cat. no. SC-2005) antibodies were purchased from Santa Cruz Biotechnology (Santa Cruz, CA, USA). Protein A-horseradish peroxidase was from Thermo Scientific (Little Chalfont, Buckinghamshire, UK).

### 2.2. Protein Extraction and Western Blot Analysis

Western blot assay was performed as previously described [43] on whole or membrane lysates. Proteins were purified from growth-arrested SH-SY5Y cells stimulated or not with 0.1 *μ*M N-fMLP, in the presence or absence of the appropriate amounts of selective inhibitors. Whole lysates were obtained by incubation with RIPA buffer (50 mM Tris-HCl, pH 7.4, 150 mM NaCl, 1% NP-40, 1 mM EDTA, 0.25% sodium deoxycholate, 1 mM NaF, 10 *μ*M Na_3_VO_4_, 1 mM phenylmethylsulfonylfluoride (PMSF), 10 *μ*g/mL aprotinin, 10 *μ*g/mL pepstatin, and 10 *μ*g/mL leupeptin) for 45 min at 4°C [44]. Membrane proteins were purified by incubating SH-SY5Y cells with a buffer containing 10 mM Tris-HCl, 1 mM CaCl_2_, 150 mM NaCl, 1 mM phenylmethylsulfonylfuoride, 10 *μ*g/mL aprotinin, 10 *μ*g/mL pepstatin, and 10 *μ*g/mL leupeptin (Buffer I). Samples were centrifuged at 400 × g for 10 minutes at 4°C, to obtain a cytosolic (supernatant) and membrane (pellet) fraction. Membrane fraction was washed three times in Buffer I and incubated overnight at 4°C in constant agitation with a buffer containing 125 mM Tris-HCl, 1 mM PMSF, 1% Triton X-100, 10 *μ*g/mL aprotinin, 10 *μ*g/mL pepstatin, and 10 *μ*g/mL leupeptin (Buffer II) [45]. Bio-Rad protein assay was used to determine protein concentration (Bio-Rad, Hercules, CA, USA). Equal amounts of proteins (40-60 *μ*g, see legends to figures) were separated on 8%, 10%, or 12% SDS-PAGE (Bio-Rad), depending on molecular weight of analyzed protein. Proteins were electroblotted onto an immobilion-P PVDF membrane (Thermo Fisher Scientific), and aspecific binding sites were blocked by incubating membranes at room temperature with a solution of 5% nonfat dry milk in Tris-buffered saline 0.1% Tween for 1 hour. After overnight incubation at 4°C with primary antibodies, membranes were washed and incubated at room temperature for 1 hour with peroxidase-conjugated mouse or rabbit IgG. The expression of targeted proteins was detected by an ECL chemiluminescence reagent kit and visualized by autoradiography. A Discover Pharmacia scanner equipped with a sun spark classic densitometric workstation was used to evaluate band densitometry. The equal amount of loaded protein was determined by reprobing the same filters with an anti-*α*-tubulin or anti-CD133 antibody. All western blot experiments are representative of at least four independent experiments.

### 2.3. Proliferation Assay

SH-SY5Y cells (4 × 10^4^) were seeded in a 24-well plate and cultured in DMEM supplemented with 15% FBS with or without 0.1 *μ*M N-fMLP, in the presence or absence of the appropriate amounts of selective inhibitors. The number of trypan blue-positive and trypan blue-negative cells was counted at 24, 48, and 72 hours, by direct counting using Burker's chamber, as previously described [46]. Five independent experiments were performed in triplicate.

### 2.4. Wound Healing Assay

Wound healing assay was performed as previously described [47]. SH-SY5Y cells were cultured until 100% confluences with DMEM containing 15% FBS, at 37°C and 5% CO_2_. The cell monolayer was scratched with an 80 *μ*m diameter sterile tip, and the plates were washed with PBS to remove the detached cells. Once the wound injury was induced, cells were serum-deprived for 24 hours and incubated with 0.1 *μ*M N-fMLP or with the vehicle, in the presence or absence of the appropriate amounts of selective inhibitors. An image was captured in the same area of the plates 0, 24, and 48 hours after the wound. Images were taken by using the Leica AF6000 Modular System and processed by using the Leica LAS AF lite software. ImageJ software was used to quantify the covered surface from four independent experiments.

### 2.5. Reactive Oxygen Species Assay

Generation of intracellular ROS was determined by measuring 2′,7′-dichlorodihydrofluorescein-diacetate (H2DCFDA; Sigma) oxidation into the fluorescent 2′,7′-dichlorofluorescein (DCF). Briefly, 4 × 10^4^ SH-SY5Y cells were seeded in a 12-well plate and cultured at 37°C, 5% CO_2_ with DMEM supplemented with 15% FBS. Cells were then serum-deprived for 24 hours and stimulated for different times with 0.1 *μ*M N-fMLP in the presence or absence of the appropriate amounts of selective inhibitors. Cells were then incubated for 45 minutes at 37°C with 50 *μ*M H2DCFDA, and the oxidization to the fluorescent DCF was analyzed on the FACS flow cytometer BD Biosciences Accuri C6 Flow Cytometer (BD Biosciences). Five independent experiments were performed in triplicate.

### 2.6. Neurite Outgrowth Assay

Neurite formation was determined by plating 10^4^ cells into wells of 12-well plates in triplicate and cultured with DMEM supplemented with 15% FBS. Cells were then incubated with 0.1 *μ*M N-fMLP or with 100 ng/mL NGF for increasing times. Five images/well were recorded and analyzed for neurite elongation after 24, 48, and 72 hours using ImageJ software plugin NeuronJ from five independent experiments. The length of neurites was measured starting from the soma in each area. Untreated cells were used as controls. The morphometric analysis was performed on the images obtained under inverted-phase-contrast microscopy (Leica AF6000 Modular System) and processed by using the Leica LAS AF light software.

### 2.7. Statistical Analysis

All the data presented are expressed as mean ± standard error mean (SEM) and are representative of three or more independent experiments. For the statistical analyses, the comparisons were made by two-way analysis of variance (ANOVA). Differences were considered significant at a value of *p* < 0.05. All the analyses were performed with GraphPad Prism version 7 (GraphPad Softweare, San Diego, CA, USA).

## 3. Results and Discussion

### 3.1. FPR1 Stimulation by N-fMLP Induces NOX2 Activation in SH-SY5Y Cells

The human neuroblastoma SH-SY5Y cell line is characterized by a catecholaminergic phenotype, since it can synthesize both dopamine and noradrenaline [48] and represents an *in vitro* model widely used in neuropsychiatric research [48–50]. NOX2 is expressed in SH-SY5Y cells [51, 52], as well as in the brain, in the microglia, astrocytes, and neurons [33], which also express NOX1 and NOX4 [33]. Induction of neuronal apoptosis in response to the brain-derived neurotrophic factor is mediated by NOX2 [53], which is also involved in long-term potentiation and learning [54, 55] and in NMDA receptor signalling [56]. Learning and memory are impaired in NOX2 and p47^phox^ knockout mice [57]. Furthermore, there is evidence for a role of microglial NOX2 in inflammatory neurodegeneration [58, 59] and in the injury of the nervous system, as demonstrated by the observation that NOX2 inhibition or knockdown improves the outcome of the spinal cord injury model in mice [60]. FPR-mediated NADPH oxidase-dependent ROS generation results also involved in the progression of Alzheimer's disease, mainly due to the activation of redox-sensitive pathways [61]. NOX2 activation requires p47^phox^ phosphorylation and its membrane translocation [33, 62].

**Figure 1:**
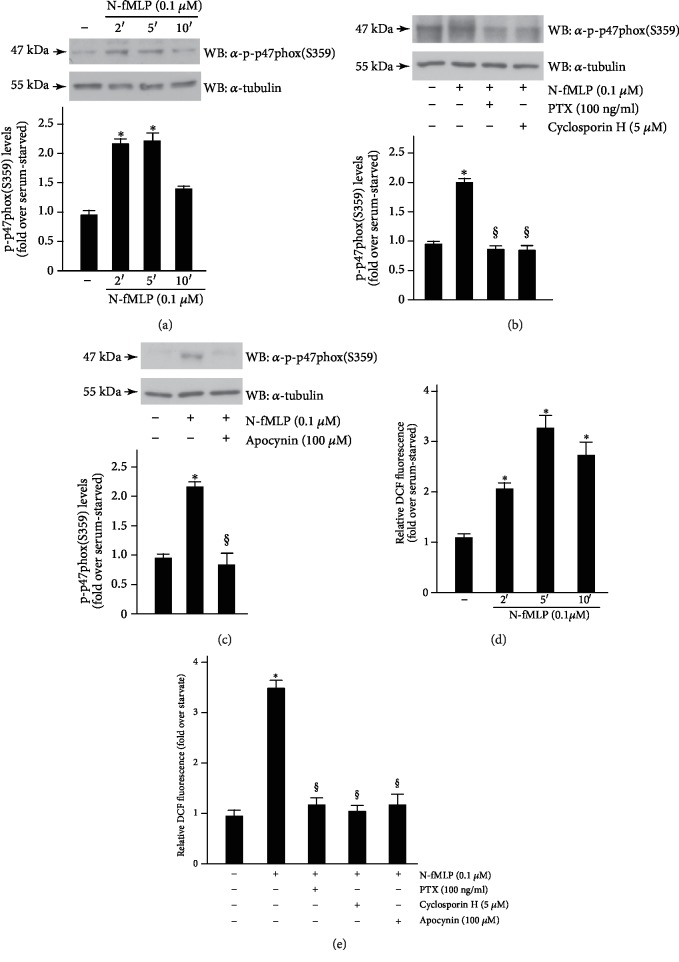
FPR1 stimulation induces NOX2 activation. SH-SY5Y cells were serum-starved for 24 hours and (a) stimulated for 2, 5, or 10 minutes with 0.1 *μ*M N-fMLP or (b) preincubated with 100 ng/mL PTX, or with 5 *μ*M cyclosporin H, or (c) with 100 *μ*M apocynin before the stimulation for 5 minutes with 0.1 *μ*M N-fMLP. Sixty micrograms of whole lysates (a, b, and c) was incubated with a phospho-p47^phox^(S359)-specific antibody (*α*-p-p47^phox^(S359)), and an anti-tubulin (*α*-tubulin) antibody was used as a control for protein loading. Band densitometry was evaluated through a scanner equipped with a densitometric workstation. Serum-starved SH-SY5Y cells were (d) stimulated with N-fMLP for increasing time or (e) preincubated with PTX, or cyclosporin H, or apocynin before the stimulation with 0.1 *μ*M N-fMLP for 5 minutes. Detection of ROS was determined by measuring the level of DCF. ^∗^*p* < 0.05 compared to unstimulated cells. ^§^*p* < 0.05 compared to N-fMLP stimulated cells.

We observed that, in SH-SY5Y cells, N-fMLP induces time-dependent phosphorylation of p47^phox^ within the first 5 min, which decreases after 10 min of stimulation (Figure 1(a)). SH-SY5Y cells were also treated with PTX, which ADP-ribosylates Gi alpha subunit conjugated to FPR1, or with cyclosporin H, a competitive antagonist of FPR1. The results show that p47^phox^ phosphorylation is completely prevented by preincubation with PTX, or cyclosporin H (Figure 1(b)), suggesting that FPR1 is crucially involved in NADPH oxidase activation. Pretreatment with apocynin (Figure 1(c)), which prevents serine phosphorylation of p47^phox^ and, in turn, NADPH oxidase activation, significantly reduces p47^phox^ phosphorylation. Accordingly, stimulation for different times with N-fMLP induces NOX2-dependent ROS generation with a maximum of ROS production occurring at 5 min (Figure 1(d)) which is prevented by preincubation with PTX, or ciclosporin H, or apocynin (Figure 1(e)).

### 3.2. FPR1 Stimulation by a Formylated Peptide Induces NOX2-Dependent TrkA Transactivation

Survival of sympathetic and sensory neurons, axon growth and synapse formation, neurotransmitter and neuropeptide synthesis [63] are mediated by NGF which binds TrkA and induces its homodimerization followed by autophosphorylation of each monomer. The NPXY and the YLDIG motif, located in the juxtamembrane region and in the C-terminus of TrkA, respectively, are then phosphorylated creating docking sited for signalling molecules [64]. Y490, Y751, and Y785 represent the main phosphotyrosine residues of TrkA in the juxtamembrane, in the tyrosine kinase, and in the intracellular C-terminal domains, respectively [5, 7].

**Figure 2:**
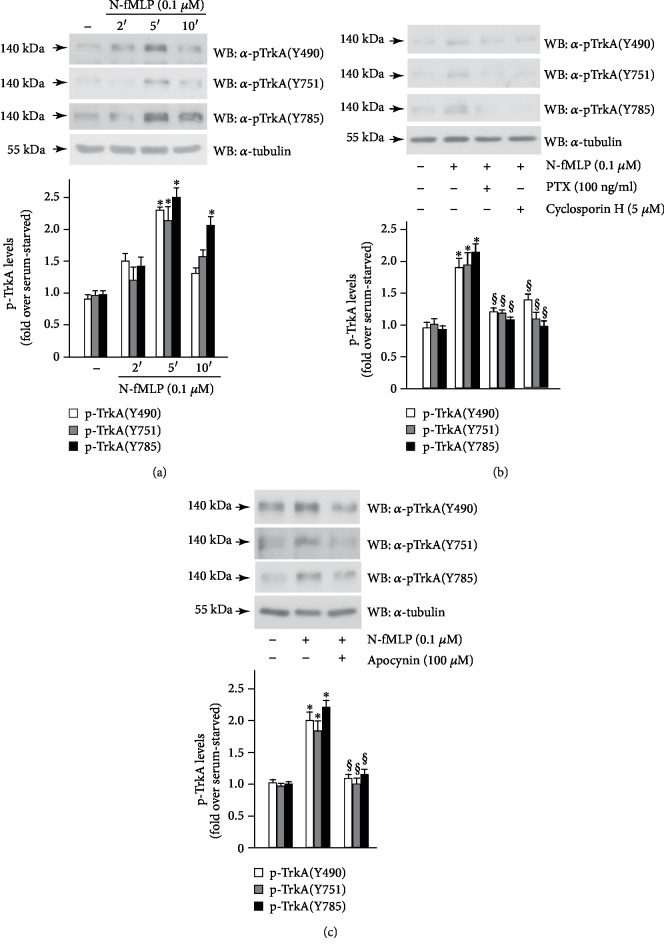
FPR1 stimulation triggers TrkA transactivation. SH-SY5Y cells were growth-arrested for 24 hours and (a) stimulated with N-fMLP for increasing time or (b) preincubated with PTX or cyclosporin H or (c) pretreated with apocynin, before the stimulation with 0.1 *μ*M N-fMLP for 5 minutes. Fifty micrograms of total protein lysates was resolved on 10% SDS-PAGE and immunoblotted with anti-phospho TrkA (Y490) (*α*-pTrkA(Y490)), or anti-phospho TrkA (Y751) (*α*-pTrkA(Y751)), or anti-phospho TrkA (Y785) (*α*-pTrkA(Y785)) antibodies. An anti-tubulin (*α*-tubulin) antibody was used as a control for protein loading. Bar graphs show the densitometric analysis performed on phosphorylated bands. All the experiments are representative of four independent experiments. ^∗^*p* < 0.05 compared to unstimulated cells. ^§^*p* < 0.05 compared to N-fMLP-stimulated cells.

Cross-communication between GPCRs and RTKs provides the connection between the wide variety of GPCRs and the strong signalling ability of RTKs to modulate intracellular pathways involved in many biological functions. SH-SY5Y cells express both FPR1 [65] and TrkA [66] receptors. We analyzed FPR1-mediated TrkA transactivation in these cells, and in time-course experiments, we observed that the incubation with 0.1 *μ*M N-fMLP elicits the phosphorylation of Y490, Y751, and Y785 residues of TrkA with the highest levels of phosphorylation occurring at 5 min (Figure 2(a)). Preincubation of SH-SY5Y cells with PTX or with cyclosporin H, before the incubation with N-fMLP, completely prevents tyrosine phosphorylation of TrkA (Figure 2(b)), strongly suggesting that TrkA transphosphorylation depends on FPR1 activation. ROS play an important role in RTK transactivation since they can inactivate, by oxidation, cysteines positioned in the catalytic site of protein tyrosine phosphatases (PTPs) [35, 36, 67]. Prevention of PTP action promotes the phosphorylated state of a RTK and, in turn, its transactivation. Several PTPs, such as NEAP/DUSP26, MEG2, SHP-1, are associated with TrkA [68–70]. We preincubated SH-SY5Y cells with apocynin before FPR1 stimulation, and we observed that N-fMLP-induced phosphorylation of Y490, Y751, and Y780 residues of TrkA is prevented (Figure 2(c)). Incubation of SH-SY5Y cells with PTX, or cyclosporin H, or apocynin, without N-fMLP stimulation, for the same times, does not affect the expression levels of TrkA (Supplementary [Supplementary-material supplementary-material-1]). These results demonstrate that NOX2-dependent ROS generation mediates the cross-talk between FPR1 and TrkA. RTK transphosphorylation can also occur via metalloprotease-mediated proteolytic cleavage of a pro-ligand, which generates a ligand able to bind and to transactivate an RTK. We cannot exclude that N-fMLP can promote the release of NGF via the activation of metalloproteases.

### 3.3. FPR1-Induced TrkA Transactivation Triggers the Ras/MAPK Pathway

Phosphorylated tyrosine 490 of TrkA provides a docking site for the Shc domain. A phosphotyrosine site on Shc recruits Grb2, which is bound to the exchange factor SOS that represents a scaffold for Ras. Activation of Ras is essential for neuronal differentiation, as well as for promoting survival of neuronal subpopulations [71], and is promoted by neurotrophin-dependent phosphorylation of RasGRF1 [72]. Active Ras triggers intracellular signalling through cRaf, PI3 kinase (PI3K), and p38MAP kinase (p38MAPK) pathways. Raf phosphorylates Mek1/2 which, in turn, phosphorylates ERK1/2 on serine and threonine residues.

**Figure 3:**
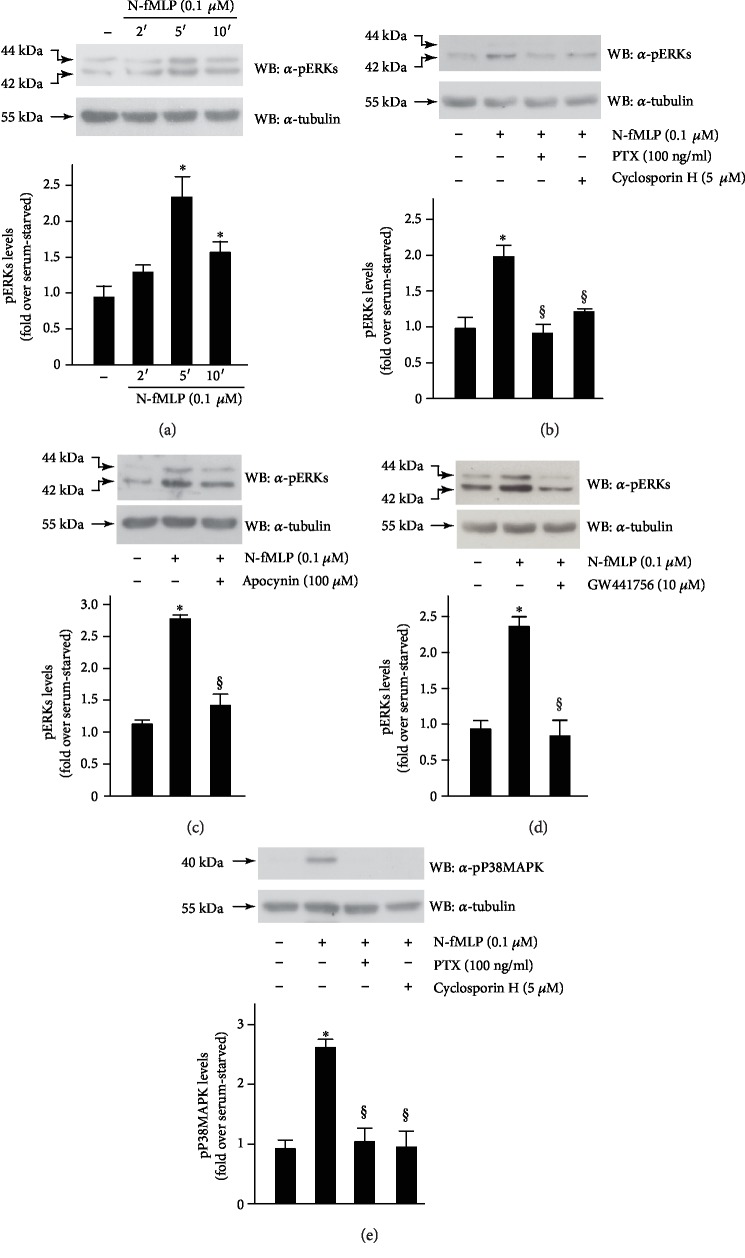
N-fMLP-induced Y490 phosphorylation is crucially involved in ERK activation. Serum-deprived SH-SY5Y cells were (a) stimulated for 2, 5, or 10 minutes with 0.1 *μ*MN-fMLP or (b and e) pretreated with PTX or cyclosporin H, or (c) with apocynin, or (d) with GW441756, before the stimulation with N-fMLP for 5 minutes. Forty micrograms of whole lysates was incubated with (a, b, c, and d) an anti-phospho-ERK (*α*-pERK) antibody or (e) with an anti-phospho-P38MAPK (*α*-pP38MAPK) antibody. An anti-tubulin (*α*-tubulin) antibody was used as a control for protein loading. The data are representative of five independent experiments. Densitometric analysis was performed as described in Materials and Methods. ^∗^*p* < 0.05 compared to unstimulated cells. ^§^*p* < 0.05 compared to N-fMLP-stimulated cells.

We analyzed ERK activation in FPR1-stimulated SH-SY5Y cells, and in western blot experiments, we observed that N-fMLP induces time-dependent phosphorylation of ERK1/2 with the maximum levels of phosphorylation occurring at 5 min (Figure 3(a)). Preincubation of SH-SY5Y cells with PTX or cyclosporin H, before N-fMLP stimulation, prevents ERK phosphorylation (Figure 3(b)). We also pretreated cells with apocynin (Figure 3(c)) or with GW441756 (Figure 3(d)), a potent and selective inhibitor of the ATP-binding site of the TrkA receptor, which in turn prevents its tyrosine phosphorylation and kinase activity. We observed that preincubation with apocynin or GW441756 before N-fMLP stimulation completely prevents ERK phosphorylation. On the other hand, incubation of unstimulated serum-deprived cells with PTX, or cyclosporin H, or apocynin, or GW441756, for the above indicated times, does not modulate the expression levels of ERKs (Supplementary [Supplementary-material supplementary-material-1]). These results suggest that N-fMLP-mediated ERK1/2 activation depends on a PTX-sensitive GPCR, NOX2-dependent ROS generation, and on FPR1-dependent TrkA transactivation.

In neurons, p38MAPK activation depends on the intracellular signalling cascade triggered by Ras-mediated binding of the exchange factor RalGDS, which results in Ral activation and Src recruitment [73]. In neuronal cells, p38MAPK can be also activated via neurotrophin-dependent activation of G proteins Rin and Rit, which belong to the Ras family [74, 75]. We observed that N-fMLP stimulation of SH-SY5Y cells for 5 min induces a significative increase of p38MAPK phosphorylation, which is prevented by preincubation with PTX or cyclosporin H (Figure 3(e)).

### 3.4. N-fMLP-Dependent Phosphorylation of Y490 and Y751 Residues of TrkA Triggers the PI3K/Akt Pathway

Phospho-Y490 of TrkA provides a recruitment site for Shc, which allows a link also for the PI3K pathway [72], and Y751 phosphorylation is essential for PI3K docking and activation [7]. PI3K generates 3-phosphate phosphoinositides, which show several effects on the development and survival of several populations of neurons. Class I of PI3Ks catalyzes *in vivo* the conversion of phosphatidylinositol (4,5)-bisphosphate into phosphatidylinositol (3,4,5)-trisphosphate. They also convert phosphatidylinositol into phosphatidylinositol 3-phosphate and phosphatidylinositol 4-phosphate into phosphatidylinositol (3,4)-bisphosphate *in vitro*. Class I of PI3Ks are activated via Ras-dependent or independent pathways [76, 77]. 3-Phosphate phosphoinositides recruit and activate phosphoinositide-dependent protein kinase 1 (PDK1) which phosphorylates and activates the serine/threonine kinase Akt.

The activity of glycogen synthase kinase 3 (GSK3) is negatively regulated by serine phosphorylation mediated by Akt [78], and, in neurons, phosphorylation-mediated GSK3 inhibition promotes the prosurvival effects induced by TrkA activation.

The PI3K/Akt/GSK3 signalling cascade may represent a diagnostic and pharmacological target for psychiatric illnesses. In human lymphocytes, PI3K levels are impaired in patients affects with schizophrenia [79] and Akt has been identified as a possible susceptibility gene for schizophrenia [80]. Furthermore, alterations of GSK3 activity represents a schizophrenia risk factor [81].

**Figure 4:**
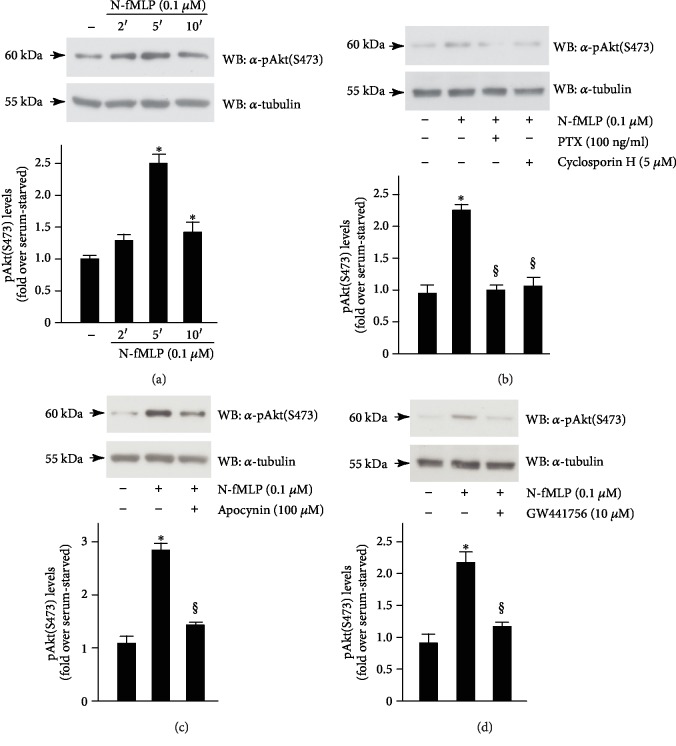
FPR1-mediated phosphorylation of Y751 residue of TrkA provides a docking site for PI3K/Akt pathway activation. SH-SY5Y cells were growth-arrested for 24 hours and (a) stimulated for 2, 5, or 10 minutes with 0.1 *μ*M N-fMLP or (b) pretreated with PTX or cyclosporin H or (c) preincubated with apocynin or (d) with GW441756, before N-fMLP stimulation. Fifty micrograms of whole lysates was immunoblotted with an anti-phospho Akt(S473) (*α*-pAkt(S473)) antibody. An anti-tubulin (*α*-tubulin) antibody was used as a control for protein loading. All the experiments are representative of five independent experiments. ^∗^*p* < 0.05 compared to unstimulated cells. ^§^*p* < 0.05 compared to N-fMLP-stimulated cells.

In SH-SY5Y cells, we observed time-dependent phosphorylation on Ser473 residue of Akt upon stimulation with N-fMLP (Figure 4(a)), which is prevented by preincubation with PTX, or with an FPR1 antagonist (Figure 4(b)). N-fMLP-induced Akt(Ser 473) phosphorylation is also hampered by a NOX2 inhibitor (Figure 4(c)) or by a TrkA inhibitor (Figure 4(d)). SH-SY5Y cells were also incubated, for the above indicated times, with PTX, or cyclosporin H, or apocynin, or GW441756 alone, and we observed that these treatments do not modulate the total expression levels of Akt (Supplementary [Supplementary-material supplementary-material-1]). Taken together, these results demonstrate that N-fMLP triggers ROS-dependent phosphorylation of Y490 and Y751 residues of TrkA which, in turn, provide docking sites for PI3K/Akt signalling.

### 3.5. FPR1-Mediated Phosphorylation of Y785 Residue of TrkA Provides a Docking Site for PLC*γ*1/PKC Pathway Activation

Following TrkA activation by NGF, PLC*γ*1 is recruited to a phosphorylated Y785 residue. TrkA mediates phosphorylation and activation of docked PLC*γ*1, which catalyzes the hydrolysis of phosphatidylinositol 4,5-bisphosphate in diacylglycerol (DAG) and inositol triphosphate (IP3). The presence of these two signalling molecules activates almost all PKC isoforms and many intracellular enzymes. In neuronal cells, PKC*δ* is required for NGF-promoted neurite outgrowth [82] and PKC*α* is one of the main targets for the regulation of genes involved in neurite [83]. In the CNS, PKC*α*, *β*, and *γ* are most extensively expressed [84] and influence neuronal signalling by short-, medium-, and long-term mechanisms [85]. In frontolimbic structures involved in mood regulation, such as hippocampus and amygdala, PKC isoenzymes are highly expressed [86] and are inhibited by lithium and valproic acid (VPA) [87]. Moreover, the regulation of processes impaired in BD, such as neuroinflammation, oxidative stress, neuroplasticity, glutamatergic neurotransmission, neurotransmitter release, and neuronal excitability involve PKC signalling [88–93].

**Figure 5:**
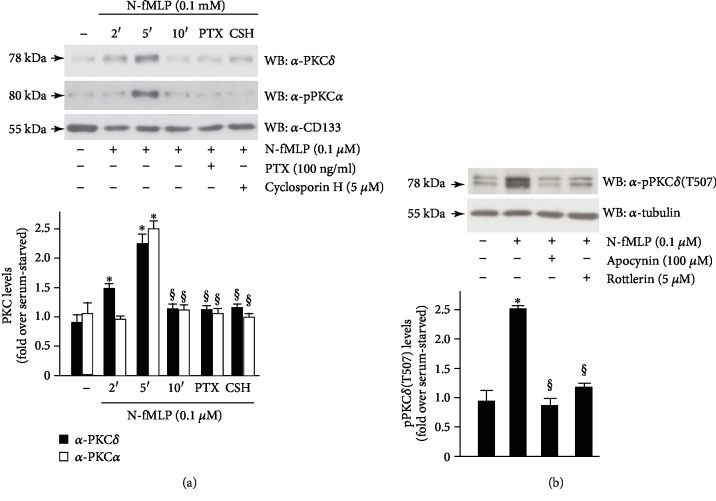
FPR1-mediated TrkA transactivation triggers PLC*γ*1/PKC pathway activation. SH-SY5Y cells were serum-deprived for 24 hours and (a) stimulated for 2, 5, or 10 minutes with N-fMLP or pretreated with PTX or cyclosporin H before the stimulation for 5 minutes with N-fMLP. (b) Cells were also preincubated with apocynin or rottlerin before N-fMLP stimulation. Fifty micrograms of membrane lysates was immunoblotted with (a) an anti-PKC*δ* (*α*-PKC*δ*) or anti-PKC*α* (*α*-PKC*α*) antibody or (b) with an anti-phosphoPKC*δ* (T507) (*α*-pPKC*δ*(T507)) antibody. An anti-CD133 (*α*-CD133) or an anti-tubulin (*α*-tubulin) antibody was used as a control for protein loading. ^∗^*p* < 0.05 compared to unstimulated cells. ^§^*p* < 0.05 compared to N-fMLP-stimulated cells.

In SH-SY5Y cells, we observed that in response to the FPR1 agonist, PKC*α* and PKC*δ* translocate to the membrane and a significant increase in their level is detectable after 5 min of exposure (Figure 5(a)). Preincubation with PTX or cyclosporin H prevents membrane translocation of PKC*α* and PKC*δ*, suggesting that it depends on FPR1 activation. Furthermore, an anti-phosphoPKC*δ*(Thr507) antibody detects PKC*δ* phosphorylation and activation in cells exposed for 5 min to N-fMLP but not in cells preincubated with apocynin or rottlerin, a specific PKC*δ* inhibitor, which prevents PKC*δ* tyrosine phosphorylation and activation (Figure 5(b)). Incubation of SH-SY5Y cells with PTX, or cyclosporin H, or apocynin, or rottlerin, without stimulation with the formylated peptide, does not affect the expression levels of PKC*α* and PKC*δ* (Supplementary [Supplementary-material supplementary-material-1]). These results indicate that PKC activation requires FPR1-mediated phosphorylation of Y785 residue of TrkA and NOX2-dependent ROS generation.

### 3.6. FPR1-Mediated TrkA Transactivation Promotes Cell Proliferation, Wound Healing, and Neurite Outgrowth

NGF stimulates growth, survival, differentiation, and maintenance of peripheral sensory and sympathetic neurons, both after injury and during development. TrkA plays a key role in neuron proliferation, differentiation, and survival in both peripheral and CNS [94, 95]. Neurotrophic factors hamper cell death, support neuronal proliferation and maturation, and improve the growth of affected neurons, as well as survival and regeneration of neurons [96, 97]. There is also increasing evidence indicating the involvement of neurotrophic factors in the survival, anti-inflammation, proliferation, and differentiation of nonneuronal tissues [98]. Overall, the systemic stimulation with NGF is related with enhanced biological activity of TrkA-expressing cells and is not connected with the induction of tumor cell proliferation [99–101].

**Figure 6:**
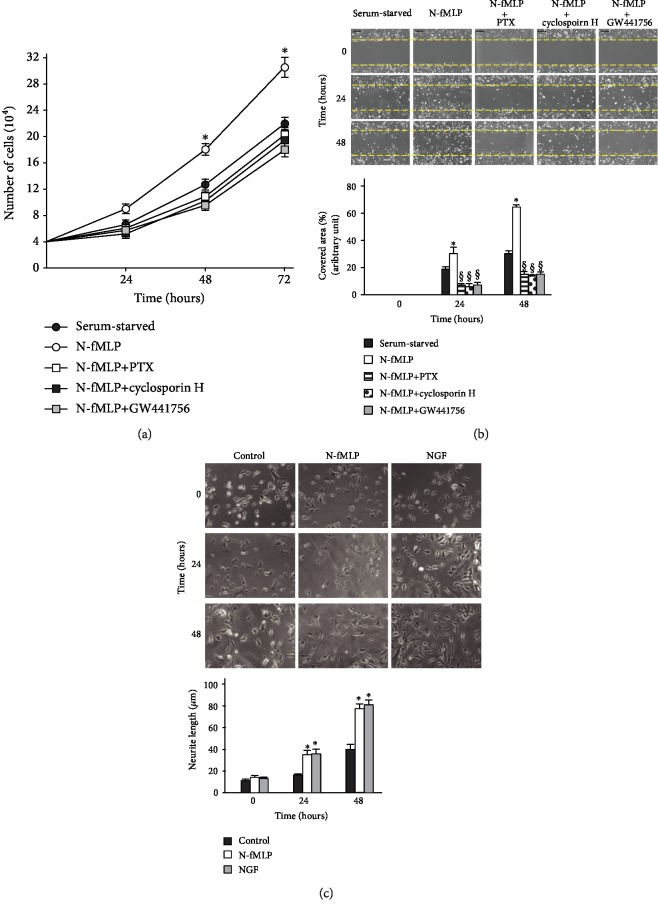
FPR1-mediated TrkA transactivation promotes SH-SY5Y cell proliferation, migration, and neurite outgrowth. (a) SH-SY5Y cells were grown in the presence or absence of 0.1 *μ*M N-fMLP and preincubated or not with PTX, or cyclosporin H, or GW441756. The cellular proliferation graph is representative of five independent experiments. Cell count was determined at 24, 48, and 72 h after plating (for all groups, 10^4^ cells/well). (b) Representative images (top) and bar graph quantization (bottom) of SH-SY5Y cell migration from 4 independent experiments. Cells were incubated with 0.1 *μ*M N-fMLP or vehicle in the presence or absence of PTX, or cyclosporin H, or GW441756. Images were acquired at 0, 24, and 48 hours after wound injury (scale bar: 20 *μ*m). ^∗^*p* < 0.05 compared to unstimulated cells. ^§^*p* < 0.05 compared to N-fMLP-stimulated cells. (c) Representative images (top) and bar graph quantization (bottom) of SH-SY5Y neurite outgrowth from five independent experiments. Neurite length was measured in untreated SH-SY5Y cells (control) or treated with 0.1 *μ*M N-fMLP (N-fMLP) or 100 ng/mL NFG (NGF) up to 48 hours. Neurite length was measured at different times (0, 24, and 48 hours). Arrows show neurite formation. ^∗^*p* < 0.05 compared to unstimulated cells. ^§^*p* < 0.05 compared to N-fMLP-stimulated cells.

We observed that N-fMLP stimulation for 24, 48, and 72 hours induces a time-dependent proliferation of SH-SY5Y serum-deprived cells (Figure 6(a)). It is prevented by preincubation with PTX, or cyclosporin H, or GW441756 suggesting that it depends on FPR1 activation and FPR1-mediated TrkA transactivation (Figure 6(a)).

NGF plays a role also in the repair process, and the pharmacological effect of NGF in accelerating wound healing was demonstrated in both normal and healing-impaired mice [102]. NGF and TrkA are differentially expressed during tissue repair, and NGF represents a bridging factor between all the cells implicated in the healing process. During tissue reorganization and wound healing, NGF might affect epithelization or contraction by priming structural or immune resident/infiltrating cells, or via the stimulation of other profibrogenic factors.

PI3K/Akt and Ras/MAPK pathways play a key role in NGF-promoted wound healing [103, 104]. These two signalling cascades are triggered by NGF-dependent phosphorylation of Y490 and Y751 residues of TrkA, which are also transphosphorylated upon FPR1 activation by N-fMLP (Figure 2(a)). FPR1 has chemotactic properties, and its function is to detect the appearance of harmful molecules, driving cells till the site of their release. Therefore, to evaluate whether FPR1 stimulation promotes wound closure, we analyzed SH-SY5Y cells in an *in vitro* wound healing assay. We observed that N-fMLP induces a more prompt cell migration after both 24 and 36 hours, compared to untreated cells (Figure 6(b)). Preincubation with PTX, or an FPR1 antagonist, or a TrkA inhibitor prevents N-fMLP-dependent wound closure (Figure 6(b)), suggesting that it depends on FPR1 activation and TrkA transactivation.

In primary cultured dorsal root ganglion cells from normal mice, resolvin D1, which efficiently binds FPR2, stimulates neurite outgrowth [105]. FPR2 also promotes neuronal differentiation, with a longer and higher number of primary neurites per cell [106], and inhibition of FPR2 signalling reduces the length of axons and dendrites, suggesting that FPR2 is involved in axonal and dendritic outgrowth [107]. NGF induces TrkA phosphorylation on a Y785 residue, which is also transphosphorylated by FPR1 activation (Figure 2(a)) and which represents a docking site for the activation of the PLC*γ*1/PKC pathway. PKC*δ* and PKC*α* are required for NGF-promoted neurite outgrowth and for the regulation of genes involved in neurite [82, 83]. Therefore, we analyzed the ability of FPR1 to promote neurite outgrowth, and we observed neurite formation after 24 and 48 hours of treatment with N-fMLP (Figure 6(c)).

## 4. Conclusions

Herein, we demonstrate that in neuroblastoma SH-SY5Y cell line, FPR1 stimulation by its agonist results in NOX2-dependent ROS generation and, in turn, in TrkA transactivation. The observation that apocynin prevents TrkA transphosphorylation and the downstream signalling cascades triggered by this receptor highlights the role of ROS in cross-talking between FPR1 and TrkA. We also demonstrate that, as a result of FPR1-mediated TrkA transactivation, phosphotyrosine Y490, Y751, and Y785 of TrkA provide docking sites for the activation of Ras/MAPK, PI3K/Akt, and PLC*γ*1/PKC signalling cascades. These promote some of the downstream responses triggered by NGF stimulation, such as cell proliferation, migration, and neurite outgrowth.

The phospho-antibodies that we used to assess TrkA phosphorylation are not specific for TrkA. In fact, the anti-phospho TrkA(Y785) can recognize also Y816 of TrkB, whereas anti-phospho TrkA(Y490) detects TrkA, TrkB, and TrkC phosphorylated isoforms. On the other hand, the anti-phospho TrkA(Y751) recognizes only TrkA. However, in many experiments, we blocked TrkA tyrosine kinase activity by using GW441756, which selectively inhibits TrkA and, in turn, TrkA signalling, (Figures 3, 4, 6(a), and 6(b)). Therefore, our results strongly suggest that TrkA is the isoform transactivated by FPR1. Nevertheless, we cannot exclude that also TrkB and TrkC, as well as other tyrosine kinase receptors, can be transactivated by FPR1 in SH-SY5Y cells. Further studies are necessary to investigate the potential contribution of other tyrosine kinase receptors in FPR1-mediated transactivation.

MAPKs play key physiological roles in the mature CNS and represent important targets for the actions of CNS-active drugs [108–110]. MAPK/ERK signalling is responsive to several drugs in the mesocorticolimbic system and is altered upon acute and chronic exposure to drugs [111]. MAPK/ERK pathway plays also an important role in fear memory reconsolidation processes, both in terms of molecular events and brain structures implicated [112]. MAPK/ERK pathway mediates many of the effects of neurotrophic factors and promotes neurite outgrowth. Accordingly, our results strongly suggest that FPR1-dependent MAPK/ERK activation promotes neurite outgrowth in SH-SY5Y cells. Mood stabilizers, such as lithium, VPA, and carbamazepine, represent a class of drugs effective in BD treatment. VPA activates the MAPK/ERK cascade [108] and induces morphological changes of human neuroblastoma cells, such as the presence of prominent growth cones and long neurites. Our results show that N-fMLP triggers the activation of FPR1/TrkA(Y490)/MAPK/ERKs cascade, suggesting that FPR1-mediated ERK activation might represent a promising therapeutical approach for BD treatment.

The regulation of PI3K/Akt/GSK3 signalling is involved in the etiology of depression and mood disorders [113, 114]. In animal models, Akt deletion elicits behaviour modifications that reflect the psychiatric appearance evocative of anxiety, schizophrenia, and depression [115]. The therapeutic effects of several psychiatric drugs are mediated by inhibition of the PI3K/Akt/GSK3 signalling. For instance, lithium, a mood stabilizer widely used for the treatment of depression, schizophrenia, and other mental illnesses, inhibits the GSK3 signalling [116]. In human lymphocytes, PI3K(p110) levels are altered in patients affected by schizophrenia [79]. On the other hand, Akt1 and Akt3 have been identified as possible susceptibility genes for schizophrenia [80] and Akt2 has been associated with anxiety- and depression-like behaviors [117]. Furthermore, Akt activity is decreased in some brain regions of major depression patients [118], and phosphorylated Akt levels are decreased in a depression animal model [119]. GPCRs and RTKs are involved in the activation of the PI3K/Akt/GSK3 pathway, and the selective activation of these receptors or GPCR-mediated RTK transactivation may be effective in treating some neuropsychiatric disorders. In SH-SY5Y cells, we observe that FPR1 activation by N-fMLP triggers TrkA(Y751) transactivation, which provides a docking site for the activation of the PI3K/Akt pathway. Phosphorylated Akt might be involved in supporting the survival of immature neurons and in contributing to cell proliferation and the initial phase of neurite outgrowth [120]. Modulation of TrkA transactivation may provide a new therapeutic strategy for the treatment of depression, schizophrenia, and other mental illnesses. Lithium, which inhibits PI3K/Akt/GSK3 signalling [116], is a mood stabilizer widely used for the treatment of these disorders. However, the molecular mechanisms of the regulation of signalling activity by lithium are poorly understood, and the identification of key signalling cascades is critical to identify novel therapeutic targets. A better comprehension of the complex PI3K/Akt/GSK3 pathway and of its activation may allow an improvement both for the diagnosis and treatments.

PKC isoenzymes are highly expressed in the brain where they play a key role in regulating pre- and postsynaptic neurotransmission. The observation that in cortical homogenates of postmortem patients affected by BD, PKC activity is increased in comparison with healthy controls [121] and that commonly used mood stabilizers inhibit PKC activity, highlight the importance of active PKC signalling in BD [122, 123]. Acute lithium treatment transiently activates PKC, whereas chronic lithium exposure results in a downregulation of PKC isoenzymes in several hippocampal structures but not in cortical and subcortical districts [110]. Neurotrophic factors regulate gene expression and synaptic plasticity via PKC signalling [124]. NGF modulates PKC activity [125], PKC influences NGF expression [126], and the interdependence of these signalling cascades modulates the fine-tuning of the synaptic strength [84]. Moreover, the activation of PKC by TrkA is required for the induction of neurite outgrowth in PC12 cells [127]. In SH-SY5Y cells, we observe that FPR1 stimulation provides a docking site in Y785 of TrkA for the binding of PLC*γ* and, in turn, for PKC activation. Membrane translocation of PKC*α* and PKC*δ*, observed in N-fMLP-stimulated SH-SY5Y cells, strongly suggests that FPR1-mediated TrkA transactivation can further support neurite outgrowth.

Oxidative stress is also implicated in BD pathophysiology [128], and scavengers of ROS show pleiotropic nonspecific PKC inhibition [129]. Quercetin administration can prevent lipid peroxidation in the prefrontal cortex, hippocampus, and striatum in a mouse model of mania induced by paradoxical sleep deprivation, as well as inhibits hyperlocomotion and oxidative stress in these districts [130]. This study provides a new aspect of the role of ROS in neuronal cells. We demonstrate that FPR1-induced ROS generation plays a key role in TrkA transactivation and, in turn, in signalling cascades triggered by this receptor, as well as in modulating cell proliferation, migration, and neurite outgrowth.

FPR1-mediated transactivation of TrkA provides further opportunities for drug discovery approaches for neuropsychiatric disorders driven by an increase of TrkA activity. The comprehension of signalling pathways responsible for TrkA transphosphorylation and of the intracellular cascades triggered by TrkA transactivation can contribute to identify new drugs efficient to interfere with targets within the FPR1 pathway. Our results also suggest that drugs able to target simultaneously FPR1 and TrkA might have enhanced therapeutic effects in neuropsychiatric disorders, compared with targeting the receptors separately.

## Data Availability

The data used to support the findings of this study are included within the article.

## Supplementary Materials

Supplementary Materials Supplementary Figure 1: representative western blot (top) and densitometric bar graph (bottom) analysis obtained from four independent experiments performed on whole lysates. SH-SY5Y cells were serum starved for 24 hours and incubated or not with PTX, cyclosporin H, apocynin, or GW441756. Lysates were immunoblotted with anti-TrkA (*α*-TrkA), or anti-Akt (*α*-Akt), or anti-PKC*α* (*α*-PKC*α*), or anti-PKC*δ* (*α*-PKC*δ*), or anti-ERKs (*α*-ERKs) antibodies. An anti-tubulin (*α*-tubulin) antibody was used as a control for protein loading.
