# Supplementary figures and images for: NOX2-Dependent Reactive Oxygen Species Regulate Formyl-Peptide Receptor 1-Mediated TrkA Transactivation in SH-SY5Y Cells
Source: Oxid Med Cell Longev. 2019 Dec 2;2019:2051235. doi: 10.1155/2019/2051235 (PMC6913242; doi:10.1155/2019/2051235)

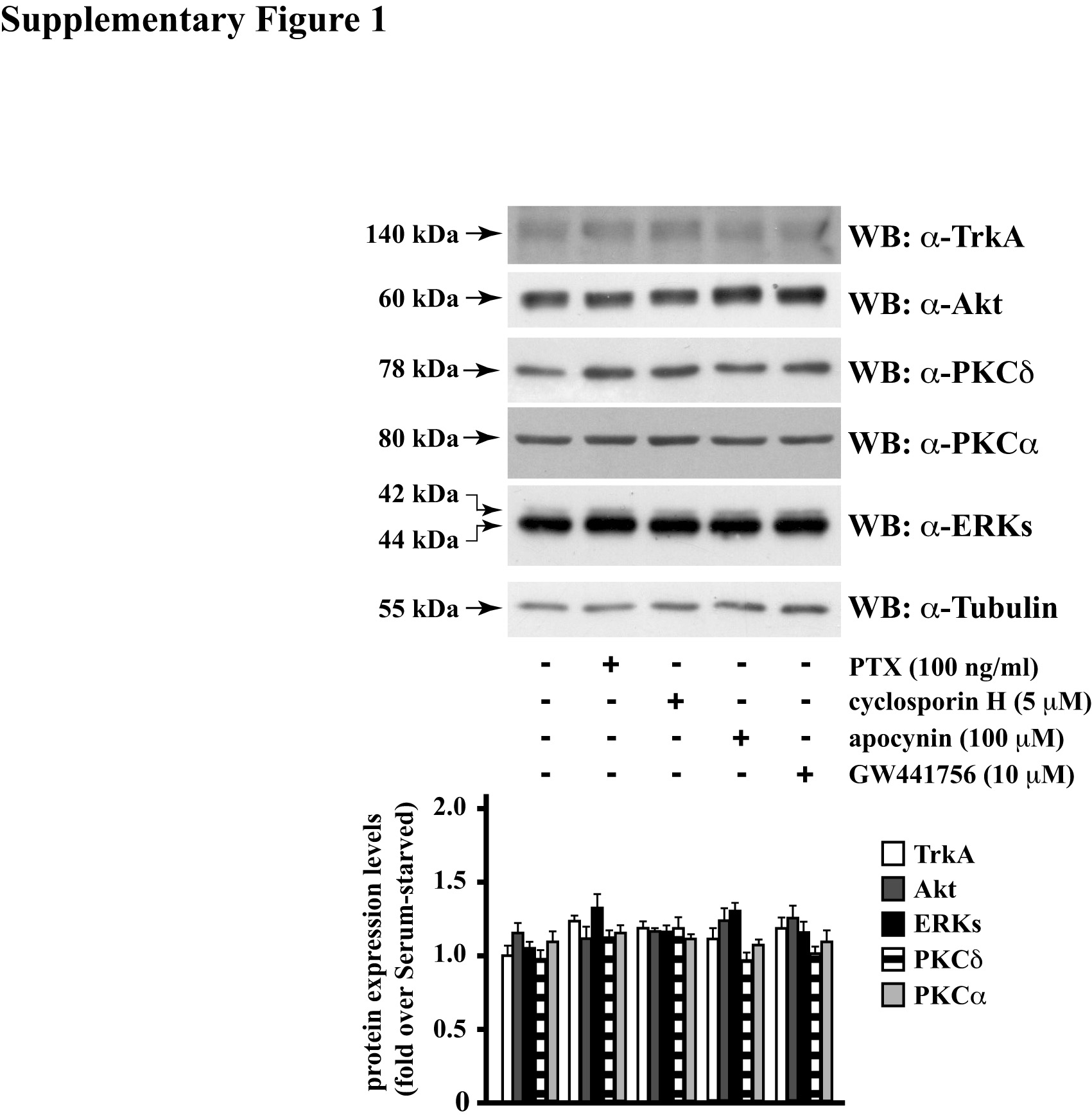

Supplement: Supplementary Materials — Supplementary Figure 1: representative western blot (top) and densitometric bar graph (bottom) analysis obtained from four independent experiments performed on whole lysates. SH-SY5Y cells were serum starved for 24 hours and incubated or not with PTX, cyclosporin H, apocynin, or GW441756. Lysates were immunoblotted with anti-TrkA (α-TrkA), or anti-Akt (α-Akt), or anti-PKCα (α-PKCα), or anti-PKCδ (α-PKCδ), or anti-ERKs (α-ERKs) antibodies. An anti-tubulin (α-tubulin) antibody was used as a control for protein loading. [file 2051235.f1.jpg]
